# Hydrostatic pressure impedes the degradation of sinking copepod carcasses and fecal pellets

**DOI:** 10.1093/plankt/fbae002

**Published:** 2024-02-01

**Authors:** Belén Franco-Cisterna, Peter Stief, Ronnie N Glud

**Affiliations:** HADAL & Nordcee, Department of Biology, University of Southern Denmark, Odense 5230, Denmark; Department of Aquatic Ecology, Netherlands Institute of Ecology (NIOO-KNAW), Wageningen 6708 PB, The Netherlands; HADAL & Nordcee, Department of Biology, University of Southern Denmark, Odense 5230, Denmark; HADAL & Nordcee, Department of Biology, University of Southern Denmark, Odense 5230, Denmark; Danish Institute for Advanced Study (DIAS), University of Southern Denmark, Odense 5230, Denmark; Department of Ocean and Environmental Sciences, Tokyo University of Marine Science and Technology, Tokyo 108-8477, Japan

**Keywords:** zooplankton, copepod, fecal pellet, carbon, hydrostatic pressure, deep sea

## Abstract

Fast-sinking zooplankton carcasses and fecal pellets appear to contribute significantly to the vertical transport of particulate organic carbon (POC), partly because of low temperature that decreases microbial degradation during the descent into the deep ocean. Increasing hydrostatic pressure could further reduce the degradation efficiency of sinking POC, but this effect remains unexplored. Here, the degradation of carcasses and fecal pellets of the abundant marine copepod *Calanus finmarchicus* was experimentally studied as a function of pressure (0.1–100 MPa). Samples were either exposed to elevated pressure in short 1-day incubations or a gradual pressure increase, simulating continuous particle sinking during a 20-day incubation. Both experiments revealed gradual inhibition of microbial respiration in the pressure range of 20–100 MPa, corresponding to 2–10-km depth. This suggests that hydrostatic pressure impedes carbon mineralization of fast-sinking carcasses and fecal pellets and enhances the deep-sea deposition rate of zooplankton-derived organic material.

## INTRODUCTION

The deep ocean (>200 m) is the most extensive habitat on our planet but is still less studied than other ecosystems (Ramirez-Llodra *et al*., 2010). Despite the darkness, low temperatures and high hydrostatic pressure, the deep ocean supports surprisingly high biodiversity (Paulus, 2021). High microbial activity has been measured in the sediment of deep-sea trenches at >6000 m (Glud *et al*., 2021), which must be sustained by labile organic matter.

The vertical sinking of particulate organic carbon (POC) from the surface ocean is considered to be the main source of organic matter fueling deep-sea communities (Ramirez-Llodra *et al*., 2010). However, the estimated organic carbon demand by mesopelagic and deeper biota exceeds by two to three orders of magnitude the vertical supply of POC (Burd *et al*., 2010; Jørgensen *et al*., 2022), suggesting unaccounted sources of carbon in the vertical fluxes. While phytoplankton aggregates and fecal pellets represent a notorious component of POC fluxes (Turner, 2015), zooplankton-derived sinking particles like carcasses, exuviae and tunicate houses are only lately recognized as significant elements of the vertical carbon flux (Henschke *et al*., 2013; Manno *et al*., 2020; Halfter *et al*., 2022; Jaspers *et al*., 2023).

The relative amount of POC exported from the surface to a given depth depends on sinking speed, the mineralization rate (i.e. aerobic respiration by heterotrophic microorganisms) and the release rates of dissolved organic carbon (DOC). The constant low temperature reduces the potential carbon mineralization rate of sinking POC in the mesopelagic (Marsay *et al*., 2015; Franco-Cisterna *et al*., 2021). Unlike temperature, hydrostatic pressure keeps increasing with depth at a rate of 10 MPa km^−1^ and may further reduce the potential mineralization rate by the inherent microbial community. However, the effect of increasing hydrostatic pressure on the degradation of zooplankton-derived sinking particles is almost unknown. The only existing study showed a reduction of fecal pellet degradation at a pressure of 15 MPa (Tamburini *et al*., 2009).

In this study, we hypothesized that increasing hydrostatic pressure up to 100 MPa reduces aerobic respiration of surface-derived, particle-attached microorganisms and thereby enhances the potential export and deposition of zooplankton-derived organic matter in the deep sea.

## METHODS

Living specimens of the copepod *Calanus finmarchicus* were obtained from the Norwegian University of Science and Technology and kept in culture at the University of Southern Denmark. Carcasses were produced by suffocation in anoxic seawater (Franco-Cisterna *et al*., 2021). Fecal pellets were produced overnight by copepods transferred to a beaker with sterile seawater and collected by pipetting the bottom within 20 h before initiating the incubations. One carcass and 40 fecal pellets were placed in 6- and 3-mL exetainers (Labco), respectively, in three replicates per pressure level. The exetainers were filled with air-saturated and sterile (0.2-μm-filtered and autoclaved) artificial seawater and were equipped with a rubber septum to ensure that ambient pressure was transmitted to the interior.

To first explore if hydrostatic pressure affected the degradation rates, we conducted short-term, 1-day experiments. For this, each set of triplicate exetainers was incubated in a rotating pressure tank (Stief *et al*., 2021). Five tanks in total were independently pressurized until 20, 40, 60, 80 and 100 MPa, and one control tank remained at atmospheric pressure (0.1 MPa; Fig. S1A). The incubations were run in darkness at 3.7°C for 24 h.

To corroborate the results of the short-term experiments and to better mimic a natural scenario, the effect of gradually increasing pressure on copepod carcass degradation was quantified over a 20-day incubation (Fig. S1B). Long-term incubations did not include fecal pellets as too few copepods were available to produce the required number of pellets (>700). Triplicates with one carcass in each exetainer were simultaneously incubated in four rotating pressure tanks in which pressure incrementally increased at a rate of 5 MPa d^−1^ (Fig. S1B), mimicking a particle descent of 500 m d^−1^, which is in the higher range of sinking velocities of *Calanus* carcasses measured *in vitro* (150–590 m d^−1^). One control tank remained at atmospheric pressure. Every 4 days, one pressure tank was opened, and the samples were retrieved for analysis. Because of the limited availability of copepods, no samples were incubated and retrieved on Day 8 (40 MPa).

In each exetainer, the oxygen concentration was discretely measured before and after pressurization. Additionally, during the 20-day incubation, oxygen was continuously measured in one exetainer at atmospheric pressure and one exetainer at increasing pressure (Stief *et al*., 2021; Fig. S1). Both discrete and continuous measurements were conducted via an optode sensor fixed to the inner glass wall and interrogated by an external optical fiber connected to a FireSting Oxygen Meter (PyroScience). Aerobic microbial respiration rates were quantified as the linear decline in oxygen concentration over time (Tables S1 and S2). For the long-term experiment, microbial respiration was converted to carbon mineralization. To this end, the oxygen consumption per pressure level was converted to oxidized carbon by using a respiratory quotient of 0.8 described for planktonic organic matter (Jørgensen *et al*., 2022). The moles of oxidized carbon were converted to mass and compared with the total carbon content of fresh carcasses (87.3 ± 28.2 μg C, *n* = 7). The latter was quantified in an elemental analyzer coupled to an isotope ratio mass spectrometer (Delta V Advantage IRMS with Thermo Scientific EA). To determine the relative pressure effect (PE) on microbial respiration, rates at high pressure were divided by the rates at atmospheric pressure. PE > 1 indicates stimulation, PE = 1 insensitivity and PE < 1 inhibition. Statistical differences in respiration rates at each high-pressure level versus atmospheric pressure were assessed with independent *t*-tests. The analyses were performed at a significance level of *α* = 0.05 in the statistical software SigmaPlot version 11.0.

## RESULTS

**Fig. 1:**
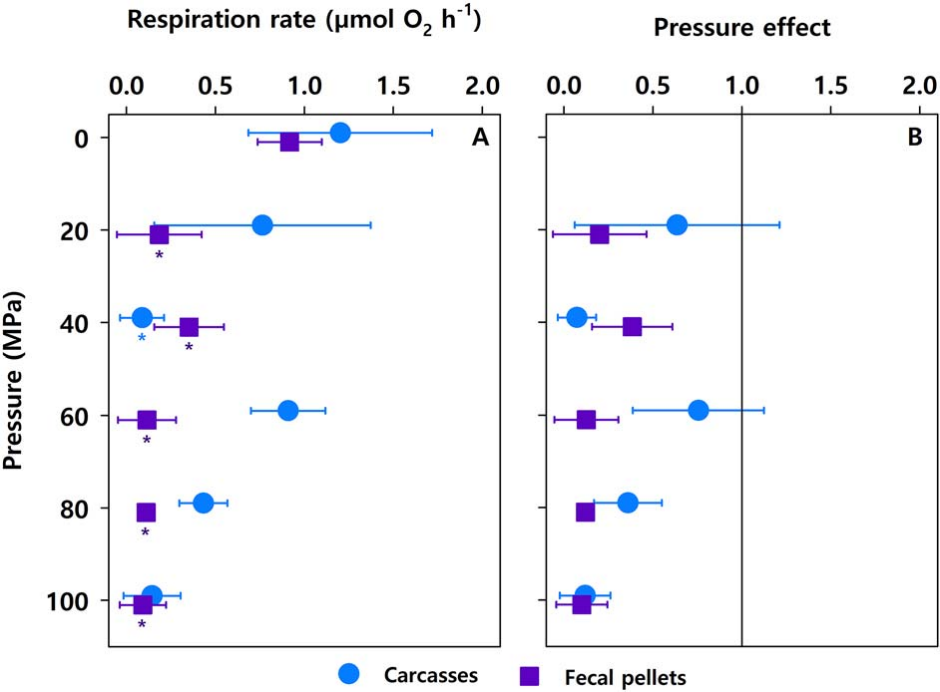
One-day pressure experiment with carcasses and fecal pellets of *Calanus finmarchicus*. (**A**) Aerobic microbial respiration rates. Asterisks indicate statistically significant differences in respiration rates between elevated and atmospheric pressure levels. (**B**) PE on aerobic microbial respiration. Values lower than 1 indicate inhibition. Means ± SD from three replicates are shown, except for fecal pellets at 80 MPa where only two replicates were incubated.

In all cases, the short-term incubations of carcasses and fecal pellets showed reduced microbial respiration rates at elevated pressures (Fig. 1A). The level of reduction showed some variations across pressures with PE values ranging from 0.1 to 0.8 but was most explicit at the highest pressure level of 100 MPa (Fig. 1B). Thus, hydrostatic pressure appeared to impede microbial respiration, which almost ceased at 100 MPa.

**Fig. 2:**
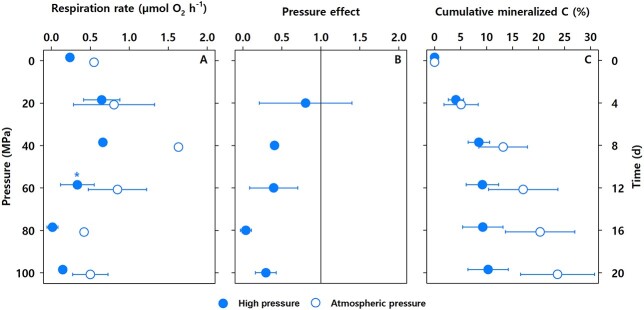
Twenty-day pressure experiment with *Calanus finmarchicus* carcasses. (**A**) Aerobic microbial respiration rates at increasing hydrostatic pressure (*y*-axis on the left) vs constant atmospheric pressure (*y*-axis on the right). Asterisks indicate statistically significant differences in respiration rates between elevated and atmospheric pressure levels. Means ± SD from three replicates are shown. Single values at 0.1 and 40 MPa, and at Days 0, 8 and 16 (atmospheric pressure) were taken from the continuous oxygen measurements. (**B**) PE on aerobic microbial respiration. Values lower than 1 indicate inhibition. (**C**) Cumulative mineralized carbon. Error bars in this panel show the propagation of uncertainty along subsequent pressure levels.

The control incubations (0.1 MPa) of the long-term experiment showed a typical, gradual increase in respiration during the first 8 days; whereafter, the respiration began to decline until the end of the experiment at Day 20 (Fig. 2A). Compared with the control, respiration rates at elevated pressures were, in all cases, reduced, most explicitly at the highest pressure levels (Fig. 2B). These results corroborate the (partial) inhibition of aerobic respiration also when hydrostatic pressure increases gradually, which mimics a natural descent of zooplankton carcass-derived POC in the ocean (Fig. 2B). Conversion of aerobic respiration to carbon mineralization rates reveals that carcasses only lost 9% of their total carbon content at high pressure compared with 20% at atmospheric pressure during the 20-day incubation (Fig. 2C). At high hydrostatic pressure, most of the carbon mineralization occurred between 0.1 and 40 MPa. At pressure levels higher than 40 MPa, the carbon in the carcasses remained rather constant. This suggests that increasing hydrostatic pressure prompts zooplanktonic organic matter preservation during the descent.

## DISCUSSION

Our study revealed partial inhibition of microbial activity associated with sinking copepod carcasses and fecal pellets at high hydrostatic pressure. Respiration rates associated with carcasses were generally higher than for fecal pellets and most noticeable in the 1-day incubation at 60 MPa. Although the investigation does not allow us to resolve the underlying reasons for these observations, there are two potential explanations. First, carcasses are more labile and degrade faster than fecal pellets (Frangoulis *et al*., 2011). Additionally, the fecal pellets incubated in this study were up to 20 h old, and we cannot exclude that some labile material could have been lost during that period (Møller *et al*., 2003). Second, if the carcass-microbiome contained a large relative abundance of piezotolerant prokaryotes, the microbial activity could have been less affected by elevated hydrostatic pressures (Stief *et al*., 2023), resulting in slightly higher respiration rates than in fecal pellet incubations. High variability in carcass-associated respiration rates per pressure level was also observed. Although only adult specimens were selected, there is a considerable fluctuation in adult sizes (Marshall *et al*., 1934), which may have induced variability in the estimated respiration rates. For future analysis, a normalization of the respiration rates by size and a higher number of replicates may reduce the data scatter.

The apparent imbalance between deep-sea carbon demand and vertical POC fluxes suggests that not all sources and mechanisms that drive carbon cycling are considered in the current carbon budgets. The importance of zooplankton for the vertical POC fluxes is increasingly being recognized since zooplankton-derived sinking particles can be found down to 3800 m (Henschke *et al*., 2013). This study reveals that increasing hydrostatic pressure consistently reduces the degradation of zooplanktonic organic matter. Our observations along with the reduction of carbon mineralization rates at low temperatures and the high sinking velocities of zooplankton-derived particles (Franco-Cisterna *et al*., 2021, 2022) likely explain the prevalence of zooplanktonic organic matter in the bathypelagic realm. However, it should be acknowledged that the resident zooplankton community and zooplankton performing diel vertical and ontogenetic migration also contribute to the active transport of carbon to the deep sea (Steinberg and Landry, 2017).

Degradation of other particles like phytoplankton aggregates also declines with increasing pressure (Tamburini *et al*., 2006; Stief *et al*., 2021, 2023), revealing a common effect of high hydrostatic pressure on organic carbon mineralization likely because of the inhibition of heterotrophic microbial activity (Tamburini *et al*., 2013; Amano *et al*., 2022). Marine snow is colonized by epipelagic, free-living bacteria and little *de novo* colonization by meso/bathypelagic microorganisms is expected (Thiele *et al*., 2015; Mestre *et al*., 2018). Epipelagic microbial communities are likely not adapted to high-pressure conditions and, thus, more prone to pressure-induced enzyme inactivation and compromised membrane integrity (Bartlett, 1992; Abe *et al*., 1999), which will reduce carbon mineralization rates. Many copepod species experience daily and seasonal vertical migration, which induces intraspecific differences in the microbiomes (Datta *et al*., 2018). Even though the depth range covered by migrating copepods is minor and pressure-associated dynamics are different than for continuously sinking particles, it remains to be tested if sinking copepod carcasses and fecal pellets produced in deeper waters undergo different degradation rates at elevated pressure.

## CONCLUSION

Our experiments revealed that high hydrostatic pressure impedes carbon mineralization of zooplanktonic organic matter. This implies that zooplankton-derived sinking particles can be exported to great depth more effectively than anticipated and fuel life in the deep ocean. Including zooplanktonic particles in the estimations of POC fluxes and the effect of high hydrostatic pressure in their mineralization will likely lessen the mismatch between carbon supply and demand in the deep sea. Future studies should address additional effects of increasing pressure on sinking zooplanktonic organic matter, such as DOC leakage and succession of the associated microbial communities.

## Supplementary Material

Franco-Cisterna_etal_supp_fbae002

## Data Availability

Data available on request from the authors.
