# Correction to: Hydrostatic pressure impedes the degradation of sinking copepod carcasses and fecal pellets

**DOI:** 10.1093/plankt/fbae008

**Published:** 2024-02-26

This is a correction to: Belén Franco-Cisterna, Peter Stief, Ronnie N Glud, Hydrostatic pressure impedes the degradation of sinking copepod carcasses and fecal pellets, *Journal of Plankton Research*, 2024; https://doi.org/10.1093/plankt/fbae002

In the originally published version of this manuscript, Figure 1 had errors and did not contain the statistically significant differences between respiration rates at high versus atmospheric pressure. Figure 1 should read:


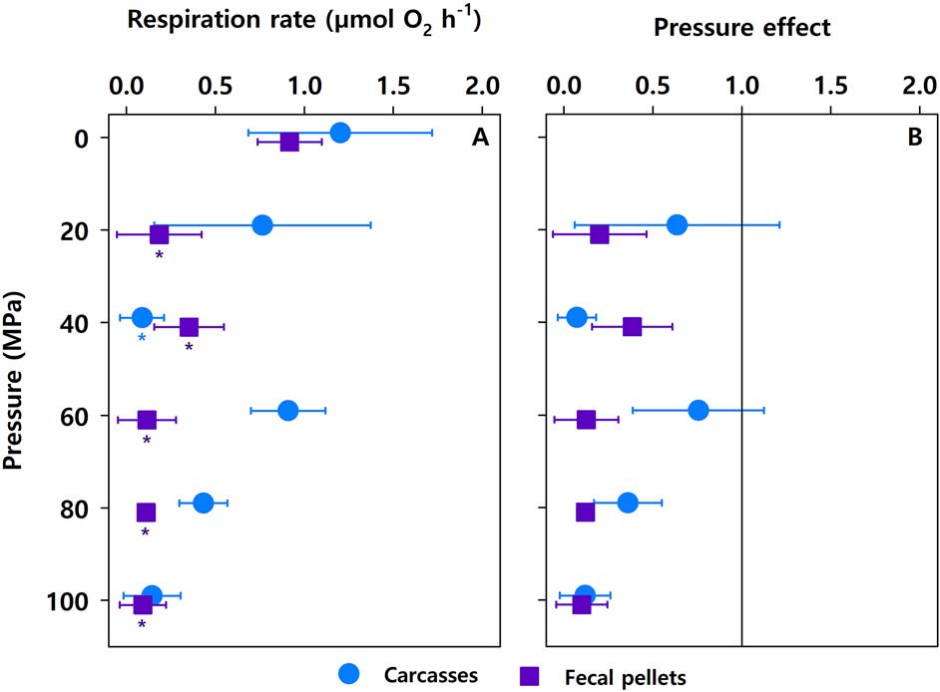


instead of:


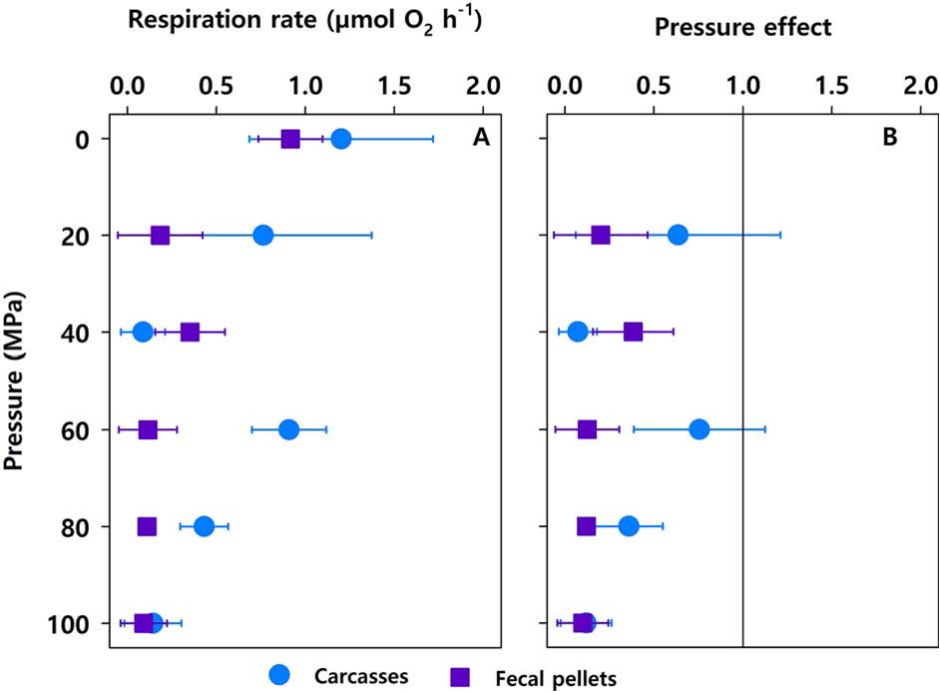


These errors have been emended in the article.

