# Supplementary material for: Hydrostatic pressure impedes the degradation of sinking copepod carcasses and fecal pellets
Source: J Plankton Res. 2024 Feb 1;46(2):219–23. doi: 10.1093/plankt/fbae002 (PMC10987097; doi:10.1093/plankt/fbae002)
Supplement: Franco-Cisterna_etal_supp_fbae002 [file franco-cisterna_etal_supp_fbae002.pdf]

Supplementary information

Hydrostatic pressure impedes the degradation of sinking copepod carcasses and fecal pellets

Belén Franco-Cisterna, Peter Stief, and Ronnie N. Glud

Table S1. Oxygen concentration resolved from discrete measurements in the incubation vials with carcasses and fecal pellets of the copepod *Calanus finmarchicus* before and after pressurization.

**Particle**	**Incubation length (d)**	**Incubation pressure (MPa)**	**Time (d)**	**Oxygen concentration ± SD (µM)**
Fecal pellets	1	0.1	0	328.4 ± 0
Fecal pellets	1	0.1	1	306.4 ± 10.2
Fecal pellets	1	20	1	324.0 ± 5.3
Fecal pellets	1	40	1	320.0 ± 5.5
Fecal pellets	1	60	1	325.6 ± 3.6
Fecal pellets	1	80	1	325.7
Fecal pellets	1	100	1	326.2 ± 2.9
Carcasses	1	0.1	0	328.4 ± 0
Carcasses	1	0.1	1	299.6 ± 16.5
Carcasses	1	20	1	310.1 ± 14.9
Carcasses	1	40	1	326.3 ± 2.7
Carcasses	1	60	1	306.6 ± 10.4
Carcasses	1	80	1	318.0 ± 5.3
Carcasses	1	100	1	325.0 ± 3.6
Carcasses	20	0.1	0	328.4 ± 0
Carcasses	20	0.1	4	234.4 ± 53.5
Carcasses	20	0.1	12	173.1 ± 1.8
Carcasses	20	0.1	20	60.6 ± 38.1
Carcasses	20	20	4	267.3 ± 25.6
Carcasses	20	60	12	190.0 ± 40.3
Carcasses	20	80	16	190.9 ± 0.5
Carcasses	20	100	20	169.6 ± 0

Table S2. Oxygen concentration resolved from continuous measurements in the incubation vials with carcasses of the copepod *Calanus finmarchicus* exposed to a gradual pressure increase.

**Particle**	**Incubation length (d)**	**Incubation pressure (MPa)**	**Time (d)**	**Oxygen concentration (µM)**
Carcasses	20	0.1	0	328.4
Carcasses	20	0.1	4	285.2
Carcasses	20	0.1	8	128.6
Carcasses	20	0.1	12	28.2
Carcasses	20	0.1	16	120.6 *
Carcasses	20	0.1	20	97.6
Carcasses	20	0.1	0	328.4
Carcasses	20	20	4	263.0
Carcasses	20	40	8	199.4
Carcasses	20	60	12	186.7
Carcasses	20	80	16	182.1
Carcasses	20	100	20	175.1

* Oxygen concentration on day 16 was higher than the previous measurement because the vial was reoxygenated on day 12.

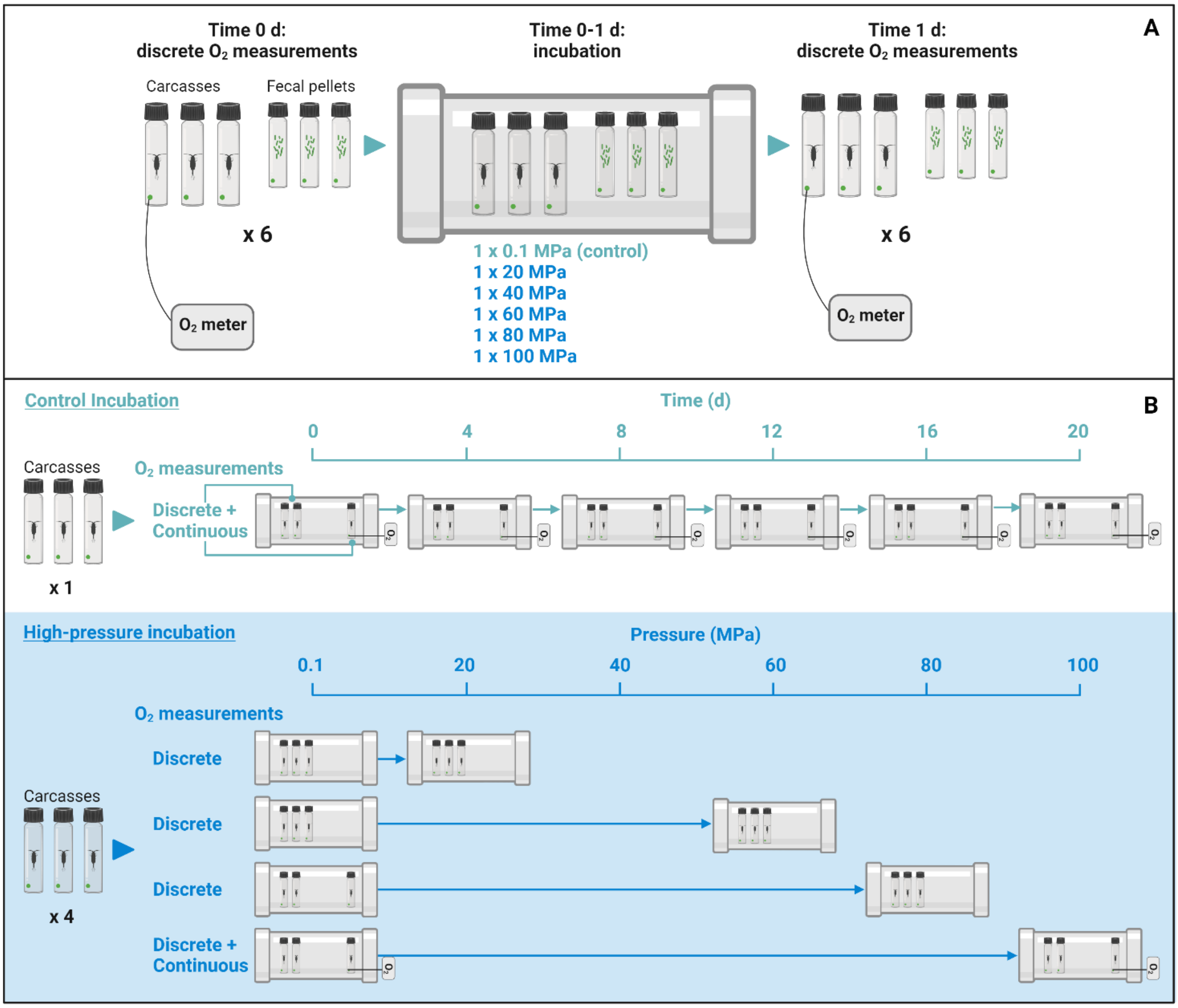

Figure S1. Schematic of the experimental design implemented in (A) one-day incubations and (B) twenty-day incubation. Created with BioRender.
